# Cloning and Functional Characterization of Two *BTB* Genes in the Predatory Mite *Metaseiulus occidentalis*

**DOI:** 10.1371/journal.pone.0144291

**Published:** 2015-12-07

**Authors:** Ke Wu, Marjorie A. Hoy

**Affiliations:** Department of Entomology and Nematology, PO Box 11620, University of Florida, Gainesville, Florida, 32611, United States of America; USDA-ARS, UNITED STATES

## Abstract

Proteins containing the BTB (Bric-à-brac, tramtrack, and Broad Complex) domain typically share low sequence similarities and are involved in a wide range of cellular functions. We previously identified two putative and closely related *BTB* genes, *BTB1* and *BTB2*, in the genome of the predatory mite *Metaseiulus occidentalis*. In the current study, full-length *BTB1* and *BTB2* cDNAs were cloned and sequenced. *BTB1* and *BTB2* encode proteins of 380 and 401 amino acids, respectively. BTB1 and BTB2 proteins each contain an N-terminal BTB domain and no other identifiable domains. Thus, they belong to a large category of BTB-domain proteins that are widely distributed in eukaryotes, yet with largely unknown function(s). *BTB1* and *BTB2* gene knockdowns in *M*. *occidentalis* females using RNAi reduced their fecundity by approximately 40% and 73%, respectively, whereas knockdown had no impact on their survival or the development of their offspring. These findings suggest these two proteins may be involved in processes related to egg production in this predatory mite, expanding the list of functions attributed to these diverse proteins.

## Introduction

The BTB domain (also known as the POZ domain) was originally identified as a motif of approximately 115 amino acids in the *Drosophila melanogaster* Bric-à-brac (Bab), tramtrack and Broad Complex transcription regulators and in many pox virus zinc finger proteins [1–4]. Proteins with the BTB domain have been found in plants, animals and viruses and typically share low levels of sequence similarity outside the domain [5].

In mammals, BTB-domain proteins participate in a variety of cellular processes including transcription regulation, cytoskeleton regulation, gating of ion channels and protein ubiquitination/degradation [6–13]. In arthropods, many BTB proteins, including *D*. *melanogaster* Fruitless (Fru) and Bab, have roles in various biological processes. The loss of the male-specific Fru protein causes impairment of mating behavior [14–16]. Paralogous Bab protein Bab1 and Bab2 are homeotic and morphogenetic regulators in the development of appendages and the abdomen in *Drosophila* [17]. Moreover, mutations in Bab1 and Bab2 result in defective ovaries, leading to impaired oogenesis [18, 19]. Interestingly, both *Bab1* and *Bab2* are expressed in *D*. *melanogaster* adult organs such as the brain and digestive tract [20], suggesting that they participate in biological processes other than development.

The BTB domain is typically found as a single copy close to the N termini of BTB proteins. It is mainly involved in protein-protein interactions with itself (self-oligomerization) or with other proteins [21, 22]. Two attributes of BTB proteins may explain why they are involved in so many functions. First, despite sharing similar secondary structures, the BTB domains from different BTB proteins show variability in their primary sequences, especially in peripheral sequences adjacent to the core BTB fold that consists of 95 amino acids [5]. This sequence variability between BTB domains allows BTB proteins to participate in a variety of protein-protein interactions [23–28]. Secondly, the presence of other protein motifs can facilitate further function differentiation in BTB proteins. For example, the kelch and BACK motifs are often found in BTB proteins involved in maintaining the stability and dynamics of actin filaments [5, 8]. DNA-binding domains, such as zinc finger or helix-turn-helix motifs, are often found in BTB proteins involved in transcriptional regulation. These proteins include many mammalian BTB proteins as well as *D*. *melanogaster* Fru and Bab proteins [5, 14, 29].

Many eukaryotic species contain a large group of BTB proteins with extended regions that consist of no recognizable motifs [5]. In vertebrates such as *Homo sapiens*, *Mus musculus* and *Danio rerio*, approximately 20% of BTB proteins belong to this category. In invertebrates, this category often represents the largest class of BTB proteins in sequenced genomes, encompassing approximately 30%, 49% and 50% of the BTB proteins in *D*. *melanogaster*, *Anopheles gambiae* and *Caenorhabditis elegans*, respectively [5]. Despite the large number of BTB proteins belonging to this category in various species, little is known about their functional significance.

We recently identified two putative *BTB* genes, *BTB1* and *BTB2*, in the genome of the predatory mite *Metaseiulus occidentalis* (= *Typhlodromus* or *Galendromus*) *occidentalis* (Nesbitt) (Arthropoda: Chelicerata: Acari: Phytoseiidae), based on homology searches using *D*. *melanogaster fruitless* as query [30]. *Metaseiulus occidentalis* is an agriculturally important biological control agent of plant-feeding pest mites such as *Tetranychus urticae* [31–34]. Previously, pesticide-resistant strains of *M*. *occidentalis* were developed through laboratory selection and were applied in biological control programs [35–37]. Further genetic improvement can benefit from studies on the molecular components such as BTB1 and BTB2 that may be involved in important biological processes.

Both *M*. *occidentalis BTB1* and *BTB2* are expressed in adult females and males, with *BTB2* showing a male-biased expression [30]. Interestingly, the predicted BTB1 (380 aa) and BTB2 (401 aa) proteins are much shorter than *D*. *melanogaster* Fru (854 aa). It should be noted that neither the *BTB1* nor the *BTB2* Gnomon gene models, created by the NCBI’s Eukaryotic Genome Annotation Pipeline, were validated by RNA seq. The NCBI’s annotation pipeline sometimes produces erroneous (e.g. partial) gene models when annotating less-conserved genes [38, 39]. Therefore, validation of software-annotated gene models is a prerequisite before commencing functional characterization of a putative gene. In the current study, we cloned and sequenced the full-length cDNAs of *BTB1* and *BTB2*. We then evaluated the domain features and phylogenetic relationship of *M*. *occidentalis* BTB1 and BTB2 proteins, and their homologs from selected species. Finally, we investigated the functional roles of these BTB proteins in adult females using an RNAi approach that produces persistent and systemic gene knockdown in *M*. *occidentalis* [40].

## Materials and Methods

### Colony sources and maintenance

The F10A inbred line was derived from the COS (Carbaryl-OP-Sulfur-resistant) colony [41, 42] by sibmating single pairs for 10 generations, as described previously [43]. The Apple Orchard (AO) colony was derived from mites collected from an organic apple orchard in Washington state where no pesticides were applied [44]. Both colonies were maintained and all experiments were performed at 22–23°C and a relative humidity (RH) of 45–55%, under a 16L:8D photoperiod. All stages of *T*. *urticae* were brushed on to paraffin-coated construction paper (75 mm × 75 mm) resting on water-soaked cotton to serve as prey.

Age-matched, mated females for the loss-of-function and quantitative reverse transcription-PCR (qRT-PCR) analyses were produced as described previously [40]. Briefly, 20 females of unknown age were collected from either the *M*. *occidentalis* F10A or AO colony and placed on pinto bean (*Phaseolus vulgaris*) leaf discs (40 mm x 60 mm) resting on water-soaked cotton that were infested with approximately 50 *T*. *urticae* females to provide prey. The *M*. *occidentalis* females, requiring feeding on *T*. *urticae* prey to produce eggs [40], were allowed to lay eggs for one day and were then removed. The eggs produced were allowed to hatch and develop. Seven days later, adult females and males emerged and were allowed to mate. Two days later, gravid (mated) females were collected individually and placed on pinto bean leaf discs (15 mm in diameter) that were infested with 4–5 *T*. *urticae* females. One day later, the *M*. *occidentalis* females produced 1–2 eggs/female, indicating that they had mated and had normally developed ovaries. They were then used for subsequent experiments.

### Cloning of the *BTB1* and *BTB2* genes

To clone the *M*. *occidentalis BTB1* and *BTB2* genes, primers were designed to amplify the bulk of *BTB1* and *BTB2* genes based on their Gnomon gene models (S1 Table). RNA from *M*. *occidental* females was extracted using RNAqueous^®^-Micro kit (Part Number Am1931, Life Technologies, CA, USA) according to the manufacturer’s instructions. cDNA for cloning was made using the cloned AMV first-strand cDNA synthesis kit (cat. no. 12328-032, Invitrogen, CA, USA) according to the manufacturer’s instructions. Nine μl of RNA isolated from a pooled sample (20) of *M*. *occidentalis* females were used in a 20-μl reverse transcription reaction containing the manufacturer’s recommended ingredients including Oligo(dT)_20_ primers. The reaction was performed in a thin-walled tube using a thermocycler (GeneAmp PCR system 9700, Applied Biosystems). The reaction was incubated at 50°C for 60 min, followed by incubation at 85°C for 5 min. PCRs were performed using 1 μl of cDNA using procedures described previously [40].

To obtain the complete cDNA sequence of the *BTB1* and *BTB2* genes, a new set of gene-specific primers matching the primers in the 5′- and 3′- Firstchoice RLM-RACE kit (cat. no. AM1700M, Ambion, Grand Island, NY, USA) were designed (S1 Table). 5’- and 3’-RACE were performed according to the manufacturer’s instructions. RNA was prepared as described above. cDNA containing appropriate adapters for 5’-and 3’-RACE was prepared as instructed by the Firstchoice RLM-RACE kit manual. Nested PCR amplifications for either 5’- or 3’-RACE were first performed with outer primer sets and then inner primer sets. All PCR reactions (in 50 μl) contained 2 μl of primers, 5 μl of 10X buffer, 4 μl of dNTPs, 0.25 μl of myTaq (cat. no. BIO-21105, Bioline USA, Taunton, MA) and 1 μl of cDNA template. The thermocycler program included the initial denaturation at 94°C for 3 min, followed by 35 cycles of denaturation at 94°C for 30 sec, annealing at 60°C for 30 sec and extension at 72°C for 30 sec. Reactions ended with a final extension of 7 min. All PCR fragments were checked by gel electrophoresis, and then cloned into pCR^™^2.1 vector (Life Technologies, Grand Island, NY, USA). Sequencing of cloned fragments was performed by the Interdisciplinary Center for Biotechnology Research (ICBR) core laboratories at the University of Florida.

### Annotation of the BTB proteins and a phylogenetic analysis

The amino acid sequences of *M*. *occidentalis* BTB1 and BTB2 proteins were used as queries to perform BLASTp searches on the GenBank database to retrieve the top hits from *Ixodes scapularis*, *Stegodyphus mimosarum*, *D*. *melanogaster*, *Nasonia vitripennis*, *Aedes aegypti*, *Homo sapiens* and *T*. *urticae* (BOGAS database). The conserved domains of the BTB proteins were identified by searching the conserved domain database of the NCBI (http://www.ncbi.nlm.nih.gov/Structure/cdd/wrpsb.cgi) using default settings [45] with their amino acid sequences. Putative nuclear localization signals in the BTB amino acid sequences were determined using cNLS Mapper (http://nls-mapper.iab.keio.ac.jp/cgi-bin/NLS_Mapper_form.cgi) [46]. An alignment of the deduced amino acid sequences of selected *BTB* genes was conducted using MAFFT 7.147 [47] with the E-INS-i alignment algorithm and the BLOSUM 62 matrix. Model selection was done with ProtTest 3.2 [48] and according to the Akaike information criterion, the LG+I+G+F model was optimum for phylogenetic analysis. Finally, a maximum likelihood analysis was performed using RAxML 7.3.2 [49], bootstrapping with 1,000 replicates.

### dsRNA synthesis and ingestion

Primers with the T7 promoter sequence added at the 5’ ends (S1 Table) were used to amplify fragments (~ 500 bp) of *BTB1* and *BTB2* genes that were later used for dsRNA synthesis. PCR products were amplified from 1 μl of cDNA prepared as described above. Bands of the expected size (~ 500 bp) were extracted and purified using a Gel Extraction kit (Qiagen, Valencia, California) according to the manufacturer’s protocol. Direct sequencing of the purified PCR products was performed at the ICBR at the University of Florida using the primers used for PCR amplification. dsRNAs were synthesized from 1 μg of purified PCR products or control template (500 bp) provided by the MEGAscript RNAi kit (cat. No. AM1626, Life Technologies, CA, USA) according to the manufacturer’s instructions. Sizes of purified dsRNAs were confirmed by gel electrophoresis in 1% agarose gel containing TBE buffer. Concentrations of purified dsRNAs were determined by a spectrophotometer (NanoDrop 1000, Thermo Scientific, USA). Purified dsRNAs were stored in elution buffer at -20°C until further use.

Ingestion of dsRNA was performed as described previously [40, 50]. Briefly, ~ 20 age-matched, mated F10A females (that had deposited 1–2 eggs each) were starved for 24 h and then placed on a parafilm disc (22 mm in diameter) resting on water-soaked cotton. Ten μl of solution containing 350 ng dsRNA/μl in 20% sucrose (Sigma) and with 3% blue food dye (McCormick, MD, USA) was applied to the parafilm disc. Both sucrose solution and blue food dye were boiled for 10 min to eliminate potential contamination by nucleases before use. F10A females were allowed to feed on the dsRNA/sucrose solution for 48 h.

Ingestion of control dsRNA at 350 ng/μl caused a small, yet significant reduction in the longevity and fecundity in AO females. In contrast, AO females fed on control dsRNA at 100 ng/μl displayed normal longevity and oviposition phenotypes when compared to TE buffer controls (S2 Table). These results suggest that, probably due to a lack of exposure to pesticides, AO females likely possess lower capacity to metabolize xenobiotics such as dsRNA than F10A females. Therefore, control and *BTB1* or *BTB2* dsRNAs were used at 100 ng/μl for feeding in subsequent experiments involving AO females. For both strains, only females with blue dye within the gastric caecae were used for further analyses. Fewer than 10% of the females were lost due to runoff and, as expected, no eggs were produced by females during starvation and dsRNA ingestion periods.

### Loss-of-function analyses

To measure the effects of *BTB1* or *BTB2* gene knockdown on viability, oviposition, embryogenesis and offspring development, mated females that had ingested control dsRNA in 20% sucrose or *BTB1* or *BTB2* dsRNA in 20% sucrose were collected individually and placed, with a male, on a bean leaf disc (22 mm in diameter) on water-soaked cotton that had been infested for 24 h with 10 *T*. *urticae* females (in order to produce prey eggs). Ingestion of prey was indicated by an increase in female body size and gradual disappearance of blue color from the gastric caecae [data not shown]. Oviposition started ~ 48 h after the spider mite diet was provided. The eggs produced by the *M*. *occidentalis* females were counted and collected every day with a fine sable-hair brush and transferred individually to a new bean leaf disc (22 mm in diameter) containing 4 *T*. *urticae* females per *M*. *occidentalis* egg. The deposited eggs were allowed to hatch and develop. The day of death for each female was recorded. The numbers and sex of developed offspring were recorded.

For the analyses of the levels of *BTB1* or *BTB2* gene knockdown, 2 days after being provided with spider mite prey, *M*. *occidentalis* females that were fed with control, *BTB1* or *BTB2* dsRNA were collected and RNA from individual females was extracted. qRT-PCR was performed subsequently to determine levels of *BTB1* or *BTB2* mRNA.

### qRT-PCR analyses

qRT-PCR analysis was performed in a similar manner as described previously [40]. The sequences for the forward and reverse primers used for the qRT-PCR are in S1 Table. The primers were validated using standard curves based on serial dilutions of cDNA to determine the primer annealing efficiencies. One no-template control was included in each experiment to check for possible contamination. qRT-PCR (in technical triplicates) was performed using conditions as previously described [40]. Four biological replicates consisting of 3 females each were performed. All results corresponded to relative quantification using the *M*. *occidentalis actin* and *GAPDH* genes as internal controls using the 2^-ΔΔCt^ method [51]. Specifically, the Ct for each target (e.g. *BTB1* gene) was subtracted by the geometric mean Ct of *actin* and *GAPDH* genes from each sample (e.g. control dsRNA or *BTB1* dsRNA) to produce ΔCt. The ΔCt from the control was then averaged to produce a mean ΔCt of the control. Then the mean ΔCt of the control was subtracted from individual ΔCt values from control or *BTB1* dsRNA treated mites, to yield the ΔΔCt. Then ΔΔCt was used to produce the 2^-ΔΔCt^ estimates. The specificity of qRT-PCR was confirmed by melting-curve analyses after each reaction. The a*ctin* and *GAPDH* genes were used as reference genes due to their demonstrated stability in *M*. *occidentalis* samples [30].

### Statistical analyses

The means and standard errors of means (SEM) were analyzed by analysis of variance (ANOVA) (JMP 8; SAS Institute, Cary, NC), and means were separated using Tukey’s HSD test (*P* < 0.05). For one-to-one comparisons, means and SEM were analyzed by ANOVA and means were separated by Student’s *t* test.

## Results and Discussion

### Cloning of the *BTB1* and *BTB2* genes

A 1,708-bp piece of the *BTB1* gene was amplified and cloned to obtain a cDNA fragment. This fragment contains the putative start and stop codons predicted by the *BTB1* Gnomon gene model. Additional 5’-UTR and 3’-UTR sequences were obtained by 5’-RACE and 3’-RACE methods, resulting in the identification of a 1,756-bp full-length cDNA (GenBank accession no. XM_003739471.1) of the *BTB1* gene. This cDNA molecule contains a 92-bp 5′-UTR, 1,143-bp ORF, and 521-bp 3′-UTR, and it encodes 380 amino acids. This result is largely in agreement with the *BTB1* Gnomon gene model, with two minor discrepancies in the 5’-UTR and 3’-UTR regions in which the Gnomon model predicts an extra 22 and 8 bases at the 5’ and 3’ ends, respectively.

Similarly, a 1,209-bp piece of the *BTB2* gene was amplified and cloned to obtain a cDNA fragment that contains the putative start codon, but not the stop codon predicted by the *BTB2* Gnomon gene model. Additional 5’-UTR sequence was obtained by 5’-RACE and the stop codon and 3’-UTR sequence were identified by 3’-RACE, resulting in the identification of a 1,800-bp full-length cDNA (GenBank accession no. XM_003746525.1) of the *BTB2* gene. This cDNA molecule contains an 84-bp 5′-UTR, 1,206-bp ORF, and 510-bp 3′-UTR, and encodes 401 amino acids. This result validates the *BTB2* Gnomon gene model in regard to the ORF region. However, there are two discrepancies between the cDNA sequencing result and the Gnomon model prediction. The *BTB2* Gnomon model predicted an extra 25 bases at the 5’-UTR region when compared to the cDNA sequence. In addition, no 3’-UTR downstream of the stop codon was predicted by the Gnomon model. In summary, cloning and sequencing of the full-length cDNAs of *BTB1* and *BTB2* genes validated and refined their Gnomon gene models. Notably, no alternative transcripts for either gene were found.

**Fig 1:**
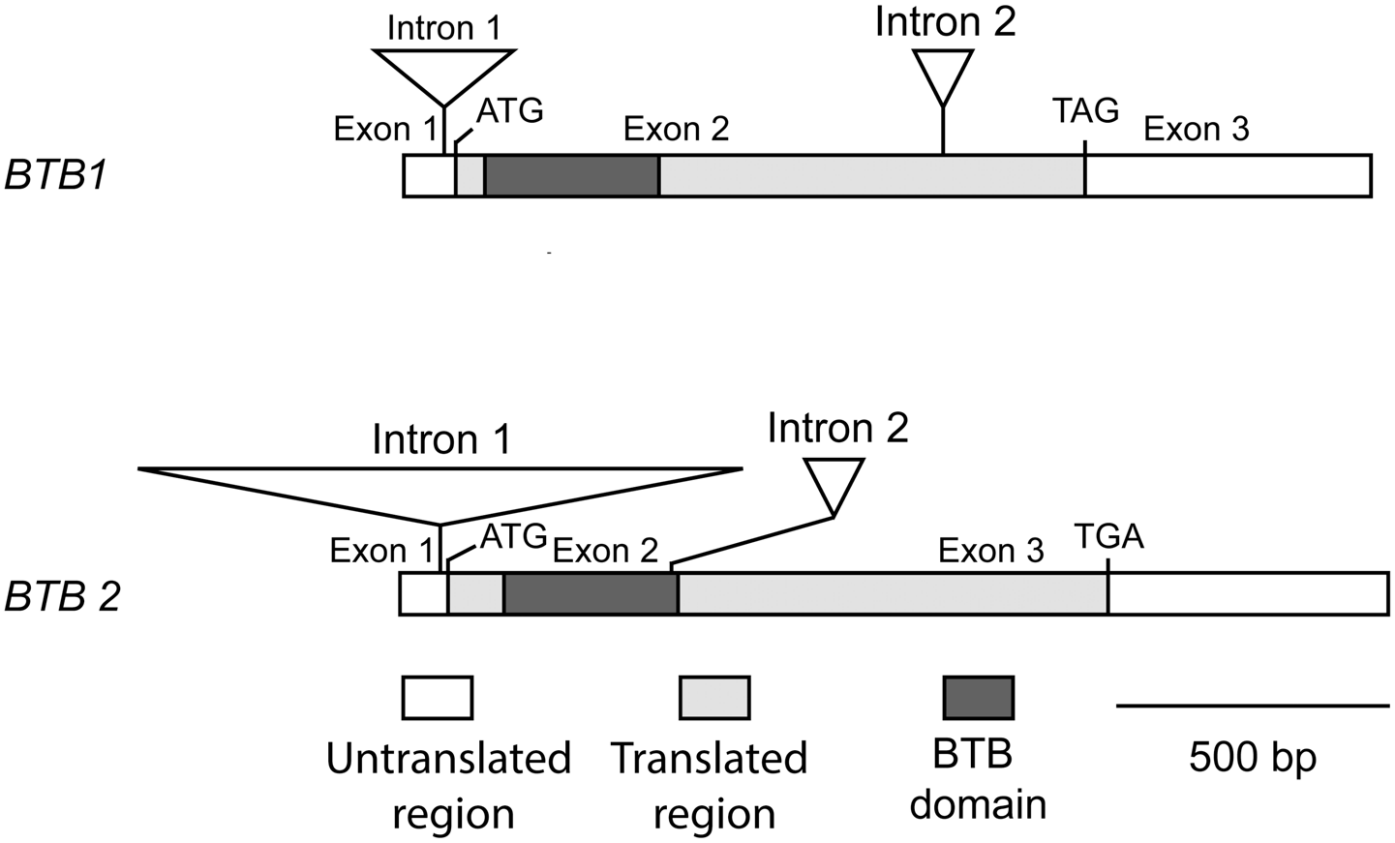
Structures of *BTB1* and *BTB2* transcripts. Untranslated and translated regions, exons and positions and sizes of introns are shown. The BTB domain is indicated.

The transcript structures of the *BTB1* and *BTB2* genes are shown in Fig 1. *BTB1* (on genome scaffold scf7180000076543) and *BTB2* (on genome scaffold scf7180000077497) span 2,113 and 2,908 bases of genomic DNA, respectively. Each gene has two introns with intron 1 of both genes located at a homologous position in the 5’-UTR. A BTB domain is located close to the N-termini of both genes. No other conserved domains were identified.

### A comparative study of the *BTB1* and *BTB2* homologs from selected species

To perform a comparative study of the putative homologs of *M*. *occidentalis BTB1* and *BTB2* genes, the deduced amino acid sequences of the *BTB1* and *BTB2* genes were used as queries to perform BLASTp searches against databases of *D*. *melanogaster*, *A*. *aegypti*, *N*. *vitripennis*, *T*. *urticae*, *I*. *scapularis*, *S*. *mimosarum*, and *H*. *sapiens*. Notably, the same top hit from each species was retrieved when either *BTB1* or *BTB2* amino acid sequences were used as BLASTp queries (S3 Table).

**Fig 2:**
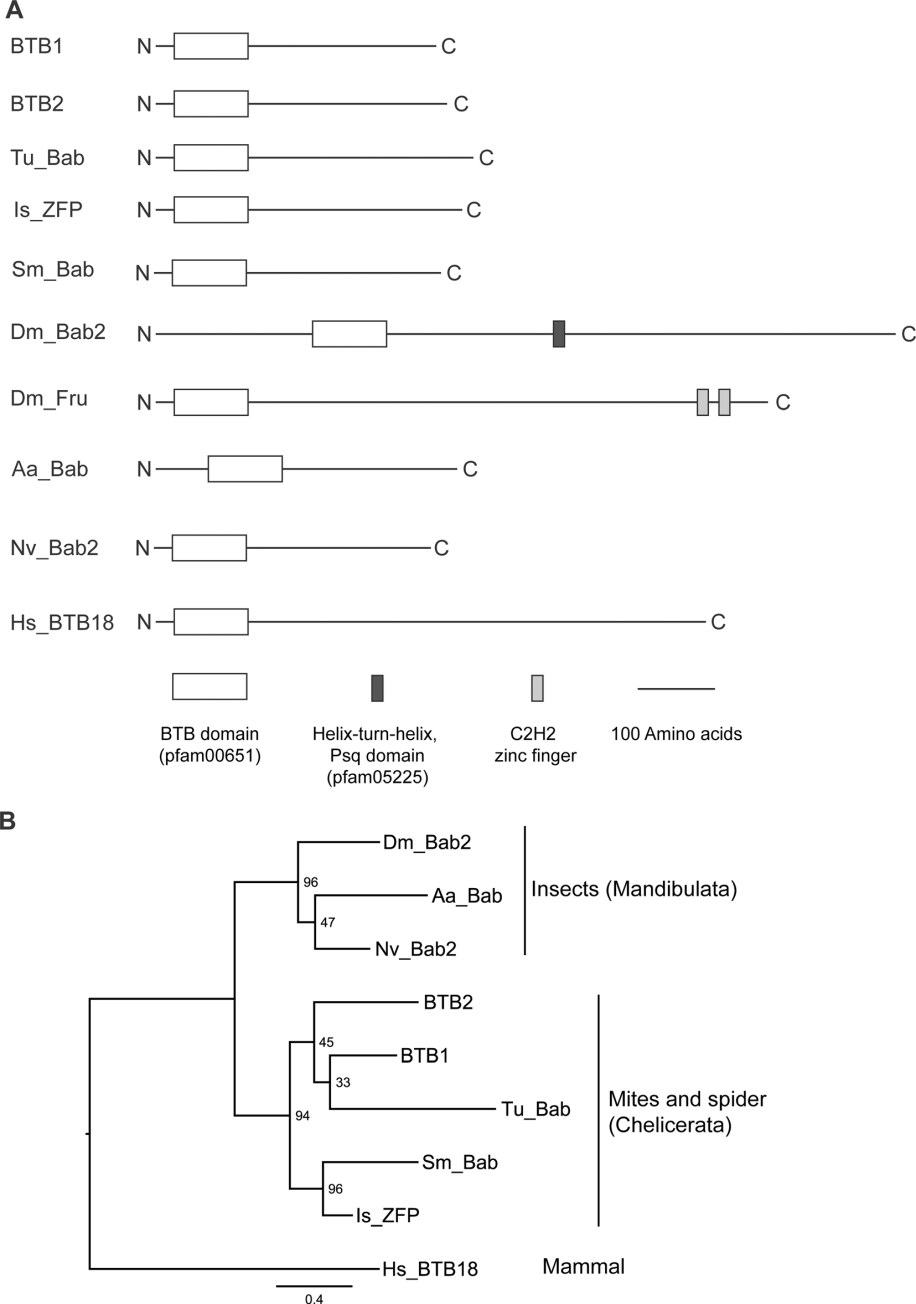
Domain structure and phylogenetic tree of BTB1 and BTB2 proteins of *M*. *occidentalis* and their closest homologs from selected species. **(A)** Schematic structures of BTB proteins from the chelicerates *M*. *occidentalis*, *T*. *urticae* (Tu), *I*. *scapularis* (Is) and *S*. *mimosarum* (Sm), the insects *D*. *melanogaster* (Dm), *Nasonia vitripennis* (Nv) and *A*. *aegypti* (Aa), and the mammal *H*. *sapiens* (Hs). Names and pfam IDs of conserved domains are shown in brackets. **(B)** A phylogenetic analysis of BTB proteins from selected species. The tree was generated using a maximum likelihood approach [49] with bootstrap support values shown at the nodes. The tree was rooted using BTB18 of the mammal *H*. *sapiens* (Hs_BTB18). The scale bar represents the numbers of substitutions per site.

Interestingly, *bab2*, but not *fruitless*, was the top hit retrieved from *D*. *melanogaster* (S3 Table), suggesting that *BTB1*/*BTB2* genes of *M*. *occidentalis* are not the *bona fide* orthologs of *fruitless* as previously thought [30]. The *BTB* genes from different species encode amino acid sequences of varying lengths (352–1,066 aa). The deduced *M*. *occidentalis* BTB1/BTB2 proteins are similar in length to their putative homologs from *T*. *urticae*, *I*. *scapularis*, *S*. *mimosarum*, *N*. *vitripennis* and *A*. *aegypti*. Fig 2A shows a comparison of the conserved domains in the BTB proteins. The BTB domains are located in a similar position (near N-termini) in all proteins. As with *M*. *occidentalis* BTB1/BTB2 proteins, no other conserved domains were found in other BTB proteins with the exception of *D*. *melanogaster* Bab2 and Fru, which possess a helix-turn-helix Psq domain and two zinc fingers, respectively (Fig 2A).

An alignment of the amino acid sequences of the *BTB1/BTB2* and their putative homologs from selected species shows that the homologous regions (even between *M*. *occidentali*s *BTB1* and *BTB2*) are limited to the BTB domains, with regions beyond the BTB domains showing only low levels of sequence similarity (S1 Fig). All BTB proteins, with the exception of *H*. *sapiens* BTB18, contain a putative nuclear localization signal (S1 Fig). Fig 2B shows the result of a phylogenetic analysis of *BTB* genes. As expected, the *BTBs* from insects (Arthropoda: Mandibulata) cluster together. *Metaseiulus occidentalis BTB1/BTB2* cluster with the *BTBs* from other chelicerates such as *T*. *urticae*, *I*. *scapularis* and *S*. *mimosarum*.

The significant differences in the domain composition and amino acid sequence length between *M*. *occidentalis BTB1*/*BTB2* and *D*. *melanogaster bab2* suggest that they may not be true orthologs either. In fact, orthologies among selected insect *bab2* homologs are weak, indicated by the varying lengths of their amino acid sequences and the lack of DNA-binding domains in all but *Drosophila* Bab2 (Fig 2 and S1 Fig). The *BTBs* in selected chelicerate arthropods are closely clustered and share similarities in both domain composition and protein length. These results suggest that these *BTBs* likely represent a lineage-specific expansion and that they may encode proteins with similar functions.

### Loss-of-function analyses

**Fig 3:**
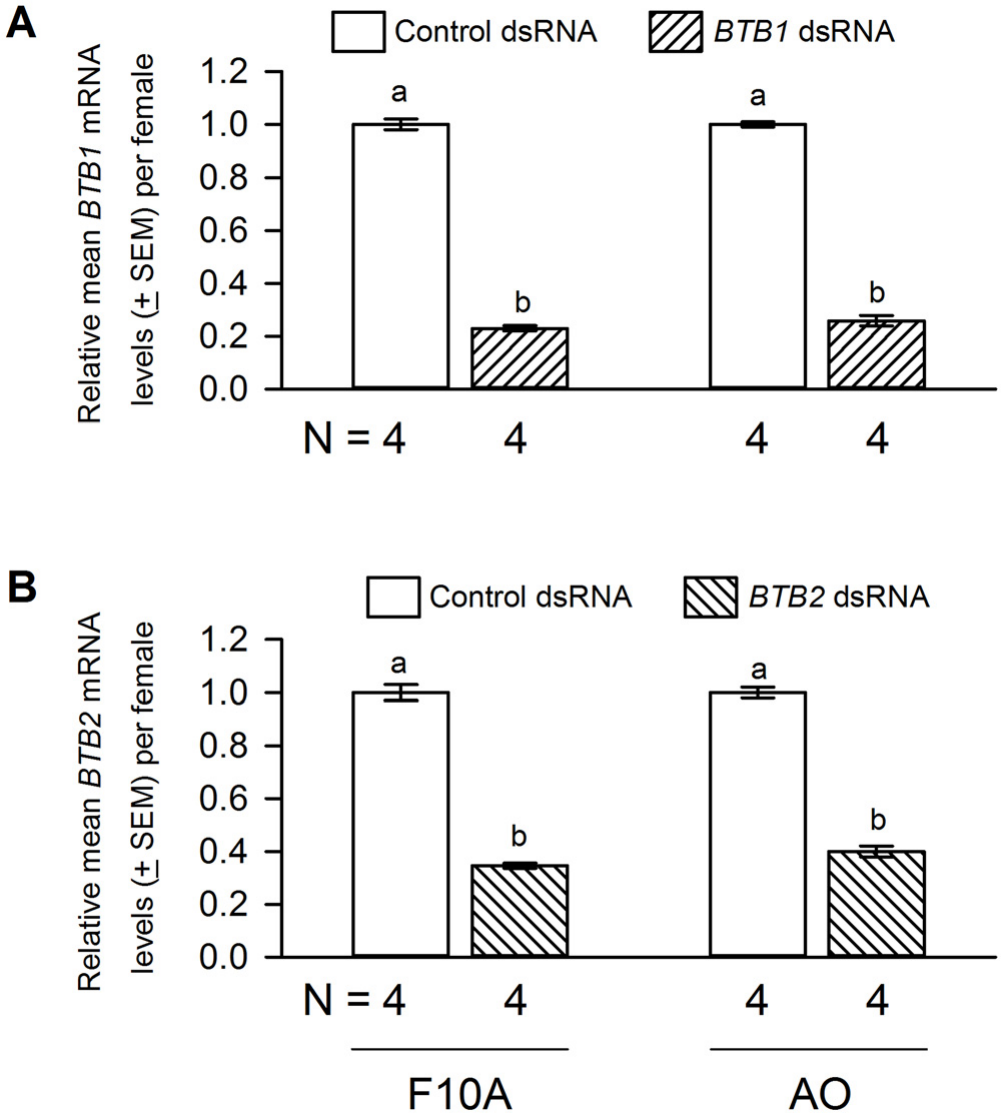
The effects of *BTB1* and *BTB2* dsRNA deliveries on the mRNA levels of *BTB1* and *BTB2*, respectively. *BTB1* **(A)** and *BTB2* **(B)** dsRNA deliveries resulted in significant knockdown in the mRNA levels of *BTB1* and *BTB2*, respectively, in either F10A or AO females. Student’s *t* test results for the comparison of the relative *BTB1* (or *BTB2*) mRNA levels in F10A (or AO) females that received control and *BTB1* dsRNA are *P* < 0.000001, 2-tailed *t* test. The *BTB1* (or *BTB2*) mRNA levels in control females were scaled to 1. Different letters denote significant differences.

**Fig 4:**
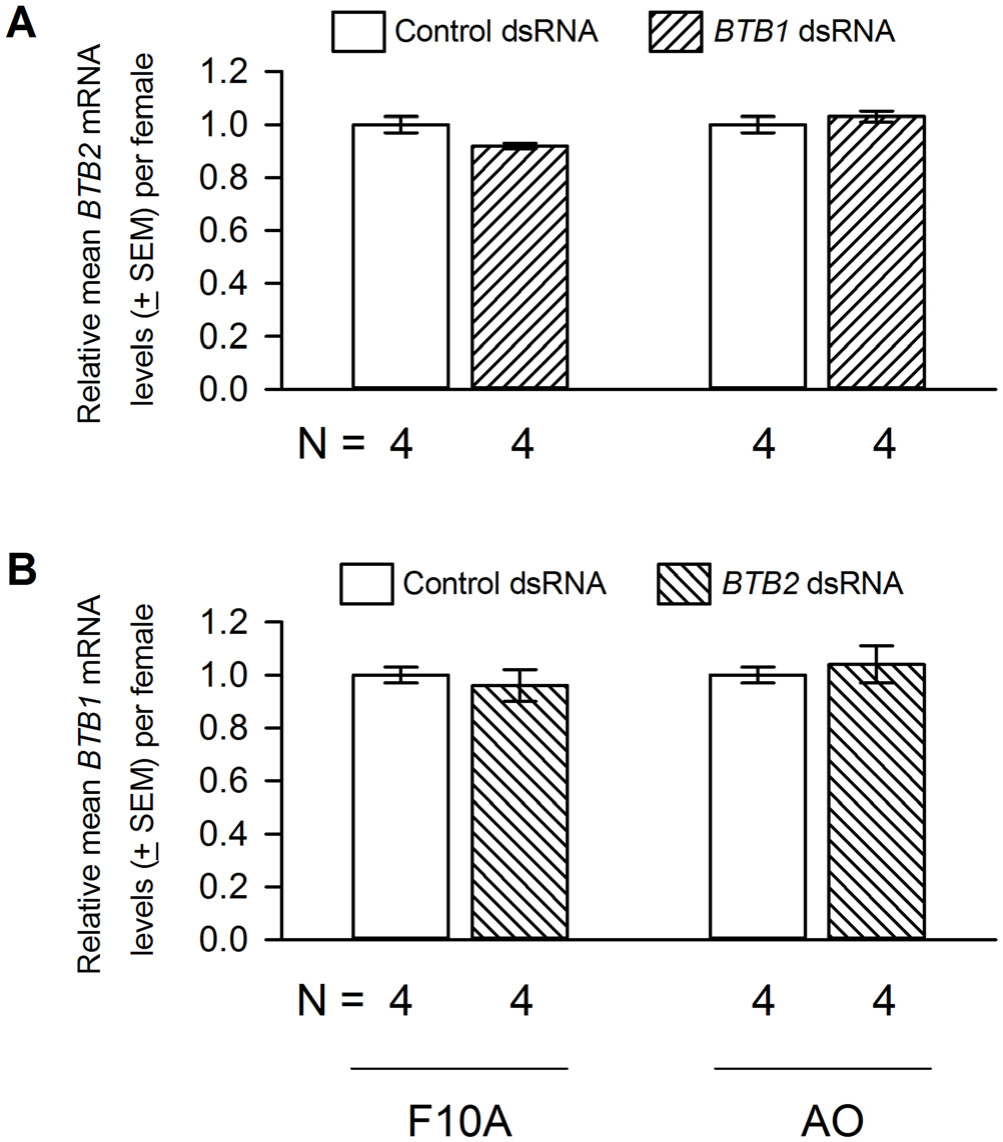
The effects of *BTB1* and *BTB2* dsRNA deliveries on the mRNA levels of *BTB2* and *BTB1*, respectively. *BTB1* **(A)** and *BTB2* **(B)** dsRNA deliveries did not alter the mRNA levels of *BTB2* and *BTB1*, respectively, in either F10A or AO females. Student’s *t* test results for the relative *BTB2* (or *BTB1*) mRNA levels in F10A (or AO) mites that received control or *BTB1* (or *BTB2*) dsRNA are *P* > 0.1, 2-tailed *t* test. The *BTB2* (or *BTB1*) mRNA levels in control females were scaled to 1.

**Fig 5:**
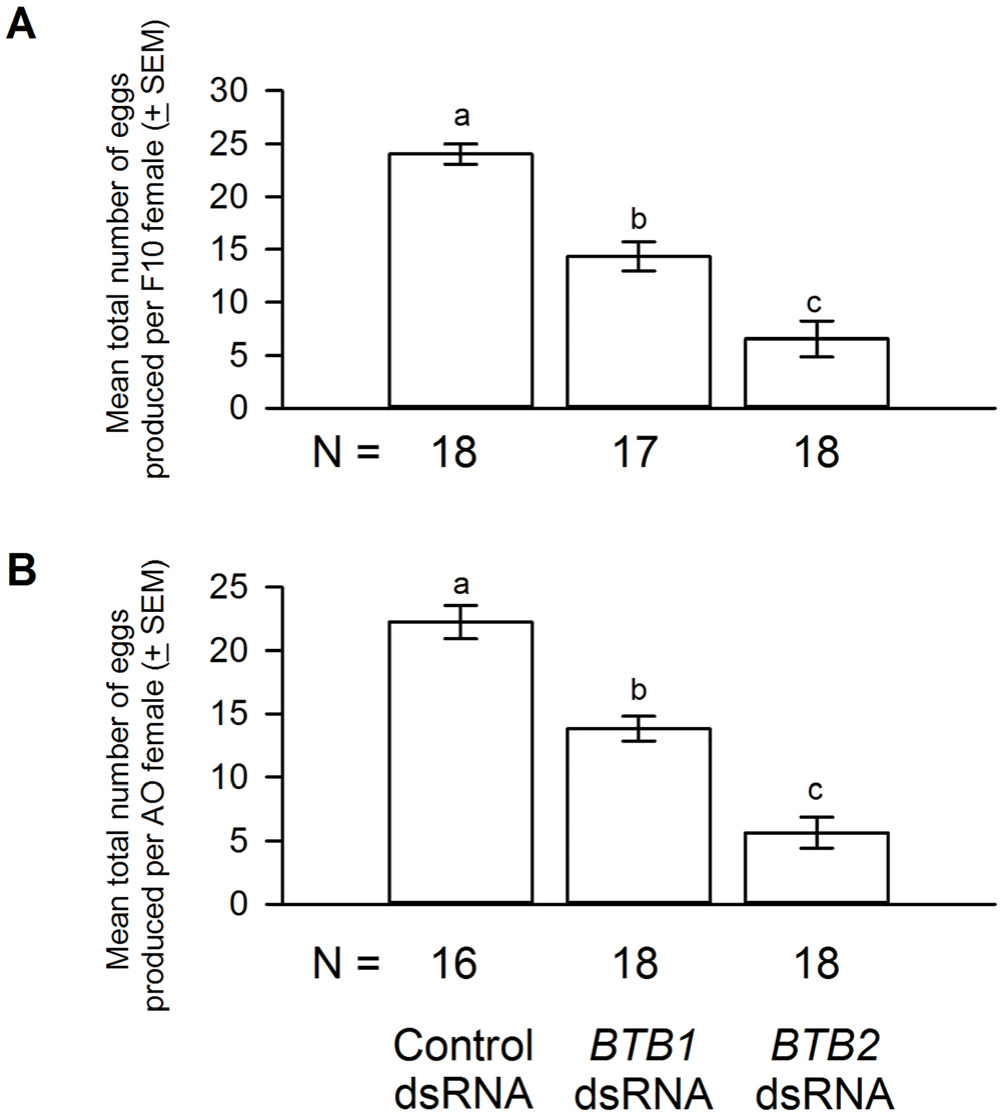
Functional effects of *BTB1* and *BTB2* dsRNA deliveries. *BTB1* and *BTB2* dsRNA deliveries significantly reduced egg production of F10A **(A)** or AO **(B)** females: One-way ANOVA, *F*_*2*,*52*_ = 41.18 and *F*_*2*,*51*_ = 48.17 for F10A and AO females, respectively; *P* < 0.0001 for both F10A and AO females, Tukey-Kramer HSD lettering for all comparisons. N represents the number of biological replicates.

Oral delivery of *BTB1* dsRNA resulted in approximately 77% and 75% reduction in the *BTB1* mRNA levels in F10A and AO *M*. *occidentalis* females, respectively (Fig 3A). In contrast, *BTB1* dsRNA delivery did not affect the *BTB2* mRNA levels (Fig 4A), suggesting that the *BTB1* gene knockdown was specific. Similar to control females, *BTB1* dsRNA-treated females appeared gravid after feeding on spider mite prey [data not shown]. However, egg production was reduced. Each F10A and AO female treated with control dsRNA produced, on average, 24.0 and 22.2 eggs, respectively. In contrast, each F10A and AO female treated with *BTB1* dsRNA produced, on average, 14.3 (a 42% reduction) and 13.8 (a 39% reduction) eggs, respectively (Fig 5). The reduction in egg laying occurred in a uniform manner over the life span of the females [data not shown]. The eggs produced by *BTB1* dsRNA-treated and control females were morphologically indistinguishable and they all hatched [data not shown], suggesting that embryogenesis was not affected in these eggs. Furthermore, the *BTB1* gene knockdown had no effect on the longevity of the females from either colony. Nor did it affect the development or sex ratios of the offspring (Table 1).

**Table 1:** Effects of *BTB1* and *BTB2* dsRNA deliveries on the survival of *M*. *occidentalis* females. Progeny development and progeny sex ratios are also compared.

Mite strain	Treatment	N	Mean days of survival ± SEM	Mean % survival of F1 progeny to adulthood ± SEM	Mean % daughters ± SEM
F10A	Control dsRNA	18	17.05 ± 0.81	84.19 ± 2.04	57.99 ± 2.17
F10A	*BTB1* dsRNA	17	15.58 ± 1.04	85.15 ± 2.43	59.75 ± 3.74
F10A	*BTB2* dsRNA	18	16.55 ± 1.29	83.05 ± 4.33	56.03 ± 5.98
AO	Control dsRNA	16	15.68 ± 0.59	84.21 ± 2.65	53.12 ± 2.22
AO	*BTB1* dsRNA	18	14.55 ± 0.88	85.66 ± 3.16	55.40 ± 4.22
AO	*BTB2* dsRNA	18	14.22 ± 0.75	86.84 ± 4.29	52.51 ± 6.42

No significant differences were found in either *BTB1* or *BTB2* dsRNA treatments on survival, offspring development or percentage of daughters (One-way ANOVA, *P* > 0.05 for all comparisons).

Fig 3B illustrates the effect of *BTB2* dsRNA delivery on the *BTB2* mRNA levels in F10A and AO females. An approximately 65% and 60% *BTB2* gene knockdown was achieved in F10A and AO females, respectively. In addition, *BTB2* dsRNA delivery had no effects on the *BTB1* mRNA levels (Fig 4B), suggesting that, similar to the *BTB1* gene knockdown, the *BTB2* gene knockdown was specific as well. In regard to egg production, each F10A and AO female treated with *BTB2* dsRNA produced, on average, 6.5 and 5.6 eggs, respectively. This represents a reduction of 73% and 75% when compared to controls (Fig 5). Furthermore, the reductions in egg production in *BTB2* dsRNA-treated F10A and AO females were significantly more severe than those in *BTB1* dsRNA-treated females (Fig 5). As with the *BTB1* gene knockdown, the decrease in egg production occurred uniformly over time. The *BTB2* gene knockdown had no effect on the morphology of the mothers and the few eggs they produced, or the hatch rates of eggs [data not shown]. Nor did it affect the survival of the females or the development and sex ratios of the few offspring produced (Table 1).

Egg production in phytoseiids is a complex process that involves oogenesis, fertilization, embryogenesis and deposition of eggs. In theory, disruption in any of the aforementioned steps may lead to the reduced egg production observed in *BTB1-* or *BTB2*-knockdown females. Notably, the egg production defect seen in *BTB1* or *BTB2* knockdown females is reminiscent of the phenotype observed in *Drosophila* strains containing mutant alleles of *bab1* and *bab2*. However, it is unclear what, if any, roles Bab1 and Bab2 may play during egg production in adult *Drosophila* females because in these mutant strains the reduced oviposition, associated with decreased oogenesis, appeared to have resulted from defective ovary formation during development [17–19]. Our gene knockdown experiments, in contrast, used mated *M*. *occidentalis* adult females that had produced 1–2 eggs before the start of the experiments, suggesting that they had functioning ovaries capable of producing oocytes that were later fertilized. Thus, our findings indicate that BTB1 and BTB2 proteins likely play a role in some aspect of egg production in *M*. *occidentalis* females.

The decrease in the numbers of eggs produced by the females treated with *BTB2*-dsRNA correlates approximately to the extent of the *BTB2*-gene knockdown (Figs 3 and 5). In contrast, the *BTB1* dsRNA treatment resulted in a less-severe reduction in egg production, despite achieving slightly higher levels of gene knockdown than the *BTB2* dsRNA treatment (Figs 3 and 5). These results suggest that BTB1 and BTB2 may be involved in distinct steps of the egg production process in which BTB1 is less critical than BTB2. Alternatively, BTB1 and BTB2 may participate at the same step of the egg production process and the variance in fecundity may be caused by a difference in their protein stability. The presence of putative nuclear localization signals in BTB1 and BTB2 suggests that they could be nuclear proteins and possibly transcription factors similar to *Drosophila* Bab1 and Bab2. Obviously, further studies are needed to pinpoint the specific processes impaired in the *BTB1* and *BTB2* dsRNA-treated females, including their cellular and subcellular localization, and the presence of possible DNA-binding motifs in these proteins.

Interestingly, both *BTB1* and *BTB2* genes are expressed in *M*. *occidentalis* males [30]. Considering BTB1/BTB2’s apparent significance in female reproduction, it would be interesting to examine any functional significance these two genes may have in males. Similarly, future studies may reveal whether these genes are involved in development like their homologs in *Drosophila* [17–19].

In summary, our study represents the first to illustrate the functional significance of two proteins belonging to a large category of seldom-investigated BTB-domain proteins. We speculate that these BTB proteins are likely involved in diverse and important biological processes, based on the large numbers of proteins in this category and the diversity in their sequences (Fig 2). Our study highlights the need, as well as the potential rewards, for further research in this class of BTB proteins.

## Supporting Information

S1 Data Data used in the current study. (XLSX)

S1 Fig A multiple sequence alignment of selected *BTB* genes. (DOCX)

S1 Table PCR primer sequences used in the current study. (DOCX)

S2 Table Determination of non-toxic control dsRNA concentrations in AO females. (DOCX)

S3 Table A list of *M*. *occidentalis* BTB1 and BTB2 proteins and their closest homologs in selected species. (DOCX)
