# Supplementary material for: Cloning and Functional Characterization of Two BTB Genes in the Predatory Mite Metaseiulus occidentalis
Source: PLoS One. 2015 Dec 7;10(12):e0144291. doi: 10.1371/journal.pone.0144291 (PMC4671623; doi:10.1371/journal.pone.0144291)
Supplement: S1 Fig — (DOCX) [file pone.0144291.s002.docx]

**S1 Fig.**

A multiple sequence alignment file (in CLUSTAL format) of the deduced amino-acid sequences of the homologs of *BTB1* and *BTB2* genes from the chelicerates *M. occidentalis*, *I. scapularis* (Is), *T. urticae* (Tu) and *S. mimosarum* (Sm), the insects *D. melanogaster* (Dm), *A. aegypti* (Aa), *N. vitripennis* (Nv) and the mammal *H. sapiens* (Hs). The amino acids composing the conserved BTB domain are shown in bold letters. The amino acids corresponding to the predicted monopartite nuclear localization signal are shown in bold and italicized letters.

BTB1 MGT---------------------------------------------------------

Is_ZFP M-----------------------------------------------------------

Sm_Bab ------------------------------------------------------------

BTB2 ------------------------------------------------------------

Dm_Bab2 MDMTKQIVDFEIKSELIGEIDQFEASDYTMAPPEEPKMVEESPQLGHLEDQNRKYSPERE

Am_Bab2 MTQQ--------------------------------------------------------

Nv_Bab2 MTQQ--------------------------------------------------------

Aa_Bab MPSD--------------------------------------------------------

Tu_Bab M-----------------------------------------------------------

Hs_BTB18 MCS---------------------------------------------------------

BTB1 ------------------------------------------------------------

Is_ZFP ------------------------------------------------------------

Sm_Bab ------------------------------------------------------------

BTB2 ------------------------------------------------------------

Dm_Bab2 VEPTLQDPSEVVDQMQKDTESVGEVKSPEKDVETELVKSKASPMNDQALTPPPRPLTSSE

Am_Bab2 ------------------------------------------------------------

Nv_Bab2 ------------------------------------------------------------

Aa_Bab -------------------------------------------------TPP--PMHSSP

Tu_Bab ------------------------------------------------------------

Hs_BTB18 ------------------------------------------------------------

BTB1 ------------------------------------------------------------

Is_ZFP ------------------------------------------------------------

Sm_Bab ------------------------------------------------------------

BTB2 ------------------------------------------------------------

Dm_Bab2 VVGLRDPEHTELRMCLEAKKSRSLPVSPQPQPNLKLAGSALFEFGQRSSPVETKIKTNPE

Am_Bab2 ------------------------------------------------------------

Nv_Bab2 ------------------------------------------------------------

Aa_Bab IMDTSDRR----------------PASVPSSSTTGLSGLSGFK----------REKFEPS

Tu_Bab ------------------------------------------------------------

Hs_BTB18 ------------------------PASP--------------------------------

BTB1 -------------NAQQFCLKWNNHQANMLTV**FDRL---LSSRSLVDVTIGCEGRQVKAH**

Is_ZFP -------------GSQQFCLKWNNHQSNMLVV**FEQL---LSNEALVDVTLACEGHSLKAH**

Sm_Bab -------------MSQQFCLKWNNHTTNMLQV**FENL---LSTEALVDVTLACDGLSLKAH**

BTB2 -----------MGSTQQFCLKWNNHQSNMLSI**FDQL---LTSEHFVDVTLACDGLSVRAH**

Dm_Bab2 TKPPRRKIVPPSGEGQQFCLRWNNYQSNLTNV**FDEL---LQSESFVDVTLSCEGHSIKAH**

Am_Bab2 ---------SGAGSPQQFCLRWNNYQTNLTNV**FDQL---LQSESFVDVTLACDGHSVKAH**

Nv_Bab2 ---------SGAGSPQQFCLRWNNYQTNLTNV**FDQL---LQSESFVDVTLACDGHSVKAH**

Aa_Bab LK-------PTLTPNQQFCLRWNNYQTNLTSV**FDQL---LQNESFVDVTLACDGKSIKAH**

Tu_Bab -------------TQQQFMLKWNNHLSNLSDV**FSNM---LISENLVDVTIACEGASLKAH**

Hs_BTB18 ---------KILYRNPRF----------LRLA**FLQLHHQQQSDVFCDVLLQAEGEAVPAH**

:* : * .: . : ** : .:* : **

BTB1 **KVVLSACSPFF-ENLFTENPCKHPIVILK--DIRYADLKALVEFMYKGEVNVVQEQLPTL**

Is_ZFP **RMVLSACSPFF-QALFVENPCQHPIVILK--DMRYMDLKAIVEFMYKGEVNVSQDQLSAL**

Sm_Bab **KMVLSACSPFF-QSLFLENPCKHPIVIMK--DMRYSDLKAIIDFMYRGEVNVSHDQLSAL**

BTB2  **KMVLSACSPFF-QSLFIQNPCEHPIVILK--DIRFVDLKALVQFMYRGEVNVSQDQLPTL**

Dm_Bab2 **KMVLSACSPYF-QALFYDNPCQHPIIIMR--DVSWSDLKALVEFMYKGEINVCQDQINPL**

Am_Bab2 **KMVLSACSPYF-QALFFDNPCQHPIVIMK--DIKWPELKAAVEFMYKGEINVSQEQIGPL**

Nv_Bab2 **KMVLSACSPYF-QALFFDNPCQHPIVIMK--DIKWPELKAAVEFMYKGEINVSQEQIGPL**

Aa_Bab **KMVLSACSPYF-QTLFFENPCQHPIIIMR--DVKWPELKAIVDFMYKGEINVSQDQIGPL**

Tu_Bab **KMILSACSPFF-QSLFMTNPCKHPIVICK--DIRFIDLKAIIDFMYTGEVNVGQEQLPSL**

Hs_BTB18 **CCILSACSPFFTERLERERPAQGGKVVLELGGLKISTLRKLVDFLYTSEMEVSQEEAQDV**

:******:* : * .*.: :: . .: *: ::*:* .*::* ::: :

BTB1 **LKTAEALKIKGLAEV**TGEGGKS-------------------DDNKPNAIVT-----QRPD

Is_ZFP **LKTAEALKVKGLAEV**TGDNRHGV---------------VSVDGADSRTIST-----ARAE

Sm_Bab **LKTAETLKVKGLAEV**TGENRQA----SGIVQQEI--TQQSVSTPTPITPSA-----QRTD

BTB2 **LKAAETLKIKGLAEV**TNESGQK----STVGGSLG----VSHNGHHSSSGGV-----TKTE

Dm_Bab2 **LKVAETLKIRGLAEV**SAGRGEG----GASALPMSAFDDEDEEEELASATAI-----LQQD

Am_Bab2 **LKVAESLKIRGLADV**NNEHELA----SRSNLEEA-----------ASAAL----------

Nv_Bab2 **LKVAESLKIRGLADV**NSEHELA----SRAGLDDS----LNL----ASAAA--------AS

Aa_Bab **LKIAEMLKIRGLADV**NGDQELNQVEHGEPSVNIADETGSNNDSNNSSTSSLLHRGIIKKE

Tu_Bab **LKAAETLKVKGLAEV**TEKQARS----------------QSAFSGSP***LYSK***----------

Hs_BTB18 **LSAARQLRVSELESL**QLEGGKL----VKAPQGRR----LNRECLQPTSAA----------

*. *. *:: * .: .

BTB1 SPGTS***SRR----KRQRVR***RKSTDSVVAHSD--SEESV-PKASRTEHDDSSMDGA------

Is_ZFP S***PTMS***-***KR***----***KRGRP***RRRSRSDSKSDSD---DQGAPPAIRIKTPESPEIIE-------

Sm_Bab TP***PLQGKR***----***KRGRP***RKRSLSDSNRSD----DEG--VAAKIKEPDSPEIEE-------

BTB2 QPSVQ---------AAPSPLSSTPAAIASN--ATHGALVAAMQQTALKAQLAAA----AQ

Dm_Bab2 G---DADP-------DEE***MKAKRPRLLPE***GVLDLN---QRQRKRSRDGSYATP-------

Am_Bab2 -------------***HRKKRRRISGE***RSPPA--CSPDRIPGSASIPDDGEPSQVGGGVIVPD

Nv_Bab2 SLQAQQQQ----***QQRKKRRLA***SGDRSPSAGGLSPD---ARASDRDEQDSASTG-----HE

Aa_Bab GRLISMDPTHNNNNNPPHMQSKKPRT------------SRERESSQISIRDFP-------

Tu_Bab ---------------***PKRRRARGE***SSKTKD-------SSCQTSESEDDSGVKR-----FH

Hs_BTB18 ---------------PISARVVTPSHH-----------PHTPLPTNQTPCPLGA------

BTB1 TSLDDGSQASLQQQSNQQQ-----SKEASINVQNNAVTVNNSSLSSVVAAG---------

Is_ZFP ----DGSLSSDRVTAVAAE------SPAARTLSSVAATLTAASSCAVAAPSN----KVAA

Sm_Bab --------------LSGDA-----SMEATSDIRSGGGPVSSNSQSQVPIHSS----NSVN

BTB2 VAQAAAAAAAVQNESRQSV-----VSAASHESNSNASTPLRQTDSPMSQ***QNRKRRRKS***AG

Dm_Bab2 SPSLQGGESEISERGSSGT-------PGQSQSQPLAMTTSTIVRNPFASPNP----QTLE

Am_Bab2 IHGMLPSSSTPRSLGSPGT--PNVSVTPQINLQELPVSL------PLPPPPP----PPP-

Nv_Bab2 SHG-LGSSSTPRSLGSPCSVMPSISVTPQINLQELPVSL------PLPPPPP----LPPP

Aa_Bab RDLSRDMSRELNAAAAAAA-------AAAAGEWPLSAALD-----TVQASTP--------

Tu_Bab ASRLGTQREKERQLLNQQK-----QLQQAE---------------ALANQQQ----QQQQ

Hs_BTB18 IRLKSLGKEEGPQENNRQN-----ADNLSGTLL--------LKRKARACPTP----QEKN

BTB1 GNNATTANTT-----TTTSNGPNSSAKDT-------DFEPTRL-LEAS-----MTTA---

Is_ZFP GLSATAANNSMSSQHTMDSVGDDTGADDA-----DFEVEPSNL-LEQS-----MTTENVP

Sm_Bab TFSKSHLSISQEPQ-----VEEQASGDEN-----EFEVEPSKL-LEQT-----LTTDNVP

BTB2 SDKGSGDENSGPESGVEDNLEETRGSDDE-SGGEEAEFEPSKL-LEQS-----MTEVRPG

Dm_Bab2 GRNSAMNAVA--NQ--RKSPAPTATGHSNGNSGAAMHSPPGGV-AVQSALPPHMAAIVPP

Am_Bab2 -------------QPGQPSSH-SIPAH---------HVPP--H-VT-S--GPHA-PV---

Nv_Bab2 GGSSSQQQQQ--QQSAQQSPHPSLLGH---------HVSPGGH-VSAT--GSHASAV---

Aa_Bab -------------K***NNRKRRWPSG***ERSSV---GSPADSTPDQH-EVPSPIPPTPSSIVQP

Tu_Bab QQQQQQEQEPEQDEDEREKIDDRNAIHQEQLTSDAIHCEASRI-LEQS-----IATASV-

Hs_BTB18 SSPSSHSQEPRENK-NDTALDPTVLSPPSLYPSVDKHLLPRKIRLSRSKPSPGICTS---

. . :

BTB1 -----DHGADNNSTDL-----------------------------------------NST

Is_ZFP VFAAVASSSSSQAAES------QQSHPKASAS-----------------DIPALVHVPAS

Sm_Bab SNSQNSTGPITLPSVSG----LEISRRSMSST-----------------DSVSQALVPAS

BTB2 HQDNNSSSSDNHTKDS-----NQAEMKPVLNF-----------------DTATGG-----

Dm_Bab2 PPSAMHHHAQQLAAQH------QLAHSHAMASALAAAAAGAGAAGAGGAGSGSGSGASAP

Am_Bab2 NHLTAH--GQQLAVQQ------QQQQQQQQQQ----------------------QQQHHP

Nv_Bab2 NHLSAHAVAQQLSVQQ------QQQQQQQQQQ----------------------QQH---

Aa_Bab ASTPTPQMPQFPIPPA-----------------LSEQMAGLSSL----------------

Tu_Bab ---VIDNGADNLIVASDVHDVAEAALKQVTLF---EEDIHQISQEDSGQFTLSTPQTSSA

Hs_BTB18 KPSSILSGSSSVPATPG----RRLWRQRSVNK-----------------------ETPED

BTB1 GPENTLIP-----------DGLDIKP-----IITLEEGGATPT-----------------

Is_ZFP LPSDISIS-----------SQLDIKPSPSSLIPPFEEQALSP------------------

Sm_Bab IPSDSLG------------SNLQGPDSPQDIKPQILSF----------------------

BTB2 -------------------NPLTINPGDPSHVLAFAAAAQAA------------------

Dm_Bab2 TGGTGVAGSGAGAAVGSHHDDMEIKPEIAEMIREEERAKMIES-------------GGHG

Am_Bab2 QPGPIVVPPNPG-------DDLEIKPGIAEMIREEERVSLFF------------------

Nv_Bab2 QPNIPVVG-----------DDLEIKPGIAEMIREEERE----------------------

Aa_Bab ---SGLAGAGSSTGAGNHPDDMEIKPGIAEMIREEERVSWIV------------------

Tu_Bab TPSSNISHQG---------RRLMMSDSHKSVLKDYLGKPISAFDVIIDDWKKSIVWQYFG

Hs_BTB18 KPKPGRA------------SPLQSTPNPSGLGKTGGSR----------------------

:

BTB1 ---GAIVPASGASSLSTGTTDAISSL----------------------------------

Is_ZFP ----AVPPQPSGSGSGDGSSGAMMMA----------------------------------

Sm_Bab ----DDPPISPVPGPSHGSERSMMM-----------------------------------

BTB2 ----QVAGAAASATPGD-------------------------------------------

Dm_Bab2 GWMGAAAAATGAASVADSYQYQLQSMWQK------CWNTNQQNLVQQLRFRERGPLKSWR

Am_Bab2 ------------------------------------------------------------

Nv_Bab2 ------------------------------------------------------------

Aa_Bab ------------------------------------------------------------

Tu_Bab G-LVYKNPETGSVSVVDSERHYCLKCIIE------CQEKNPDEIFERC------------

Hs_BTB18 ----KRSPEVRAPNSDSAEEGQVGRVKLRKIVNGTCWEVVQ-------------------

BTB1 --------FPQGTG------------------------ATALPGTSY-------------

Is_ZFP --------FTDMSG------------------------VPAIAGPSS-------------

Sm_Bab --------YMDQSG------------------------VASIPGPSN-------------

BTB2 ----------------------------------------SVAGPSQ-------------

Dm_Bab2 PEAMAEAIFSVLKEGLSLSQAARKFDIPYPTFVLYANRVHNMLGPSLDGGADPRPKARGR

Am_Bab2 -----HFLLSKG----------------------------NIFGVGF-------------

Nv_Bab2 ------------------------------------------------------------

Aa_Bab -----ECLFS----------------------------VVSIMHQGN-------------

Tu_Bab ----NICFLSTGTA----------------------------------------------

Hs_BTB18 -----ETPLKNTQD------------------------SPQIPDPGGD------------

BTB1 ---------------------------------------QASPQSHGTY-----------

Is_ZFP -------------------------YHP---------DNQQSTPSHGRYSHP--------

Sm_Bab -------------------------YQD----------NSALVPHD--------------

BTB2 -------------------------------------------PTHGTLKL---------

Dm_Bab2 PQRILLGMWPEELIRSVIKAVVFRDYREIK--EDMSAHQYANGQGHGTYIGGGTTTNGYH

Am_Bab2 --------W---------------------------------------------------

Nv_Bab2 ------------------------------------------------------------

Aa_Bab ------------------------------------------------------------

Tu_Bab ------------------------------------TGNHKNHLRHRHQVSD--------

Hs_BTB18 -------------------------FQEPSGTQPFSSNEQEMSPTRTELCQD--------

BTB1 ------------------------------------------------------------

Is_ZFP --------------------SP--------------------------------------

Sm_Bab ------------------------------------------------------------

BTB2 ------------------------------------------------------------

Dm_Bab2 SAAAAKLAAQNAALAPPDAGSPLSSMTETLRRQILSQQQQHQQHHQQQAHHQQQPSHHQQ

Am_Bab2 ------------------------------------------------------------

Nv_Bab2 ------------------------------------------------------------

Aa_Bab ------------------------------------------------------------

Tu_Bab --------------------EPITT-----------------------------------

Hs_BTB18 --------------------SPMCTKLQ----DILVSASHSPDHPVVKSEFESSPELVEK

BTB1 --------------RAALMREP--------------------------------------

Is_ZFP ---------SP--------SAP------------TT------------------------

Sm_Bab -------------------SQP--------------------------------------

BTB2 ------------------------------------------------------------

Dm_Bab2 Q--------SPHAQSMNMYKSPAYLQR----------------------SEIEDQVSAAA

Am_Bab2 -------------------KSPRRRNE---------------------------------

Nv_Bab2 ------------------------------------------------------------

Aa_Bab ------------------------------------------------------------

Tu_Bab ---------TPSTSKSSSSKQPANKMS---------------------------------

Hs_BTB18 EPMLAIDCREPYAFDTALLEQPCEAEEYRITSAAATSELEEILDFMLCGSDIEPPIGSLE

BTB1 ------------------------------------------------EIRSL-------

Is_ZFP ------------------------------------------------PERAARFLPSTL

Sm_Bab ------------------------------------------------Q-----------

BTB2 ------------------------------------------------SADGSERL----

Dm_Bab2 AVAAAAAK----------------------------------HQQQQGERRGSENLPD-L

Am_Bab2 ------------------------------------------------QRRGRGSR----

Nv_Bab2 ------------------------------------------------RERGR-------

Aa_Bab --------------------------------------------------KGVKMV----

Tu_Bab ------------------------------------------------RKRATKVI----

Hs_BTB18 SPGAEGCRTPTYHLTETGKNWIEGEEWCLPDMELWPRELTELEKEPAGENRGPTEL---L

BTB1 ------------------------------------------------------------

Is_ZFP HCL---------------------------------------------------------

Sm_Bab ------------------------------------------------------------

BTB2 ------------------------------------------------------------

Dm_Bab2 SALGLMGLPGLNVMPSRGSGGGSGGA--APNSAASYARELSRERERDRERERERELSRQY

Am_Bab2 ------------------------------------------------------------

Nv_Bab2 ------------------------------------------------------------

Aa_Bab ------------------------------------------------------------

Tu_Bab ------------------------------------------------------------

Hs_BTB18 SPL---------VMPSEVSEVLSVGGRWTPDLEITSSQPLDGQEDKLLHVSSLDTPQRSY

BTB1 ----------VSCRLA--------------------------------------------

Is_ZFP -------LCKVGCGDR--------------------------------------------

Sm_Bab ------------------------------------------------------------

BTB2 ---------HVRSGF---------------------------------------------

Dm_Bab2 GSQSR--GSSSGSGSAKSLTASQR--PGAASPYSAAHYAKHQASAYNKRFLESLPA----

Am_Bab2 ------------------------------------------------------------

Nv_Bab2 ------------------------------------------------------------

Aa_Bab ------------------------------------------------------------

Tu_Bab ------------------------------------------------------------

Hs_BTB18 GDLSPPCSNWVETGLEVSLTTDELLYP---SPKAGKEVSGH------SELLGSLPASSEE

BTB1 ------------------------------------------------------------

Is_ZFP -SVQAAFFHCC-------------------------------------------------

Sm_Bab ------------------------------------------------------------

BTB2 ------------------------------------------------------------

Dm_Bab2 -GIDLEAFA-NGLLQKSVNKSPRFEDFFPGPGQDMSELFANPDASAAAAAAAYAPPGAIR

Am_Bab2 ------------------------------------------------------------

Nv_Bab2 ------------------------------------------------------------

Aa_Bab ------------------------------------------------------------

Tu_Bab -SISLDSFT---------------------------------------------------

Hs_BTB18 EEIDVVDWTAEGRLVPTTVPS-----VWPDPSSE--------------------------

BTB1 ---------------------

Is_ZFP ---------------------

Sm_Bab ---------------------

BTB2 ---------------------

Dm_Bab2 ESPLMKIKLEQQHATELPHED

Am_Bab2 ---------------------

Nv_Bab2 ------------FQ-------

Aa_Bab ---MVSIK-------------

Tu_Bab ---------------------

Hs_BTB18 ------------SETEVDILT
