# Supplementary material for: Cloning and Functional Characterization of Two BTB Genes in the Predatory Mite Metaseiulus occidentalis
Source: PLoS One. 2015 Dec 7;10(12):e0144291. doi: 10.1371/journal.pone.0144291 (PMC4671623; doi:10.1371/journal.pone.0144291)
Supplement: S1 Table — (DOCX) [file pone.0144291.s003.docx]

**S1 Table**. PCR primer sequences used in the current study.

| Primer set | Primer sequence |
| --- | --- |
| Cloning primer | (BTB1-F) 5’-CAGAATACGCGAGTCAATCTACC-3’  (BTB1-R) 5’-GGTGACCACAAACCCTCAA-3’  (BTB2-F) 5’-TACCCTCGGTTCTTCTCAGT-3’  (BTB2-R) 5’-AGCTGAGAGTTTCAACGTACC-3’ |
| 5’-RACE primer | (5’RACE outer) 5’-GCTGATGGCGATGAATGAACACTG-3’  (5’RACE inner) 5’-CGCGGATCCGAACACTGCGTTTGCTGGCTTTGATG-3’  (BTB1-5’outer) 5’-TCCTGAACCACATTCACCTCAC-3’  (BTB1-5’inner) 5’-TCGGCGTAGCGAATATCTTTCA-3’  (BTB2-5’outer) 5’-CGATTTCTGGCCTGATTCGTTTG-3’  (BTB2-5’inner) 5’-ACAGTGTCGGTAGTTGGTCTTG-3’ |
| 3’-RACE primer | 3’RACE outer) 5’-GCGAGCACAGAATTAATACGACT-3’  (3’RACEinner) 5’-CGCGGATCCGAATTAATACGACTCACTATAGG-3’  (BTB1-3’outer) 5’-GGTCTGATCTCGGGTGTTTCAA-3’  (BTB1-3’inner) 5’-CGGTGTGGAAACCTCTTTCACTA-3’  (BTB2-3’outer) 5’-CATGTCGCAACAGAACAGGAAG-3’  (BTB2-3’inner) 5’-GGAAAGCGGCGTCGAAGATAA-3’ |
| qRT-PCR | (actin-Frt) 5’-ACATCAAGGAGAAGCTCTGC -3’  (actin-Rrt) 5’-CCTCGGGACAACGGAAAC-3’  (GAPDH-Frt) 5’-CCGTTCATCGATGTCACCTA-3’  (GAPDH-Rrt) 5’-GATGTTGGTGGGGTCAGACT -3’  (BTB1-Frt) 5’- ATCATCACGCTGGAAGAAGG-3’  (BTB1-Rrt) 5’- GAGGGAAGAGCGAAGAGATTG-3’  (BTB2-Frt) 5’-AAGATCAAAGGTCTCGCAGAA-3’  (BTB2-Rrt) 5’-GACGACGAATGGTGTCCATTAT-3’ |
| dsRNA synthesis* | (BTB1-dsF) 5’-TAATACGACTCACTATAGGGAGGTACAAGGGTGAGGTGA  ATGT-3’  (BTB1-dsR) 5’-TAATACGACTCACTATAGGGAGGAGTTCAGATCCGTCGA  GTTATT-3’  (BTB2-dsF) 5’-TAATACGACTCACTATAGGGAGCAAACGAATCAGGCCAGA  AAT-3’  (BTB2-dsR) 5’-TAATACGACTCACTATAGGGAGTTCGAACTCGGCTTCTTCT  C-3’ |

*T7 polymerase promoter sequence (**TAATACGACTCACTATAGGGAG**) was added to 5’ end of the oligonucleotide sequence to facilitate subsequent dsRNA transcription as described in manufacturer’s protocol (MEGAScript, Ambion).
