# Supplementary material for: Cloning and Functional Characterization of Two BTB Genes in the Predatory Mite Metaseiulus occidentalis
Source: PLoS One. 2015 Dec 7;10(12):e0144291. doi: 10.1371/journal.pone.0144291 (PMC4671623; doi:10.1371/journal.pone.0144291)
Supplement: S2 Table — (DOCX) [file pone.0144291.s004.docx]

**S2 Table**. Determination of non-toxic control dsRNA concentrations in AO females.

| Treatment received by AO females | N | Mean total number of eggs produced per AO female ± SEM | Mean day of survival ± SEM |
| --- | --- | --- | --- |
| TE buffer | 10 | 21.60 ± 1.82 A | 16.20 ± 0.90 A |
| Control dsRNA at 100 ng/µl | 9 | 20.66 ± 1.35 A | 15.33 ± 0.47 A |
| Control dsRNA at 350 ng/µl | 10 | 15.50 ± 0.80 B | 11.50 ± 1.09 B |

One-way ANOVA: *F_2,28_* = 8.14 and *P* = 0.001 for days of survival; *F_2,28_* = 5.66 and *P* = 0.009 for number of eggs, Tukey-Kramer HSD lettering for all comparisons.
