# Supplementary material for: Cloning and Functional Characterization of Two BTB Genes in the Predatory Mite Metaseiulus occidentalis
Source: PLoS One. 2015 Dec 7;10(12):e0144291. doi: 10.1371/journal.pone.0144291 (PMC4671623; doi:10.1371/journal.pone.0144291)
Supplement: S3 Table — (DOCX) [file pone.0144291.s005.docx]

**S3 Table.** A list of *M. occidentalis* BTB1 and BTB2 proteins and their closest homologs in selected species.

| Species | GenBank accession number/  annotation | Protein length  (in amino acids) | BLASTp E-values obtained using BTB1 /BTB2 as queries |
| --- | --- | --- | --- |
| *Metaseiulus occidentalis* | XP_003739519.1/  BTB1 | 380 | 0/  7e-65 |
| *Metaseiulus occidentalis* | XP_003746573.1/  BTB2 | 401 | 5e-65/  0 |
| *Tetranychus urticae* | tetur22g01140*/  Bab | 470 | 1e-48/  2e-52 |
| *Ixodes scapularis* | XP_002402860.1/  ZFP | 448 | 4e-67/  2e-63 |
| *Stegodyphus mimosarum* | KFM71876.1/  Bab | 390 | 7e-62/  2e-65 |
| *Drosophila melanogaster* | NP_001261242.1/  Bab2 | 1066 | 1e-53/  2e-55 |
| *Drosophila melanogaster* | NP_732348.1/  Fru^#^ | 854 | 4e-43/  7e-42 |
| *Nasonia vitripennis* | XP_008215830.1/  Bab2 | 352 | 1e-54/  3e-59 |
| *Aedes aegypti* | XP_001660259.1/  Bab | 429 | 1e-53/  2e-57 |
| *Homo sapiens* | NP_001138573.1/  BTB18 | 712 | 1e-11/  8e-10 |

*Identifier for BOGAS database (http://bioinformatics.psb.ugent.be/orcae/overview/Tetur). Closest homologs from each species were identified as the top hits in BLASTp searches using *M. occidentalis* BTB1 and BTB2 as queries.

^#^ *Drosophila melanogaster* Fru was used as a query in a preliminary BLASTp search of the *M. occidentalis* genome that identified BTB1 and BTB2 [1].

Reference

1. Pomerantz AF, Hoy MA (2015) Expression analysis of *Drosophila doublesex*, *transformer-2*, *intersex*, *fruitless-like*, and *vitellogenin* homologs in the parahaploid predator *Metaseiulus occidentalis* (Chelicerata: Acari: Phytoseiidae). Exp Appl Acarol 65: 1-16.
